# Costs Associated with the Treatment of Clostridioides Difficile Infections

**DOI:** 10.3390/ijerph18147647

**Published:** 2021-07-19

**Authors:** Aleksandra Sierocka, Zofia Kiersnowska, Ewelina Lemiech-Mirowska, Michał Marczak

**Affiliations:** 1 Department of Management and Logistics in Healthcare, Medical University of Lodz, 90-419 Lodz, Poland; zofiakiersnowska.p@gmail.com (Z.K.); ewelina.l.mirowska@wihe.pl (E.L.-M.); michal.marczak@umed.lodz.pl (M.M.); 2 Laboratory of Epidemiology, Military Institute of Hygiene and Epidemiology (WIHE), 01-163 Warsaw, Poland

**Keywords:** infection, *Clostridioides difficile*, costs, CDI

## Abstract

Background: *Clostridioides difficile*, as the main cause of infectious diarrhoea in hospitalised patients, is a considerable challenge for medical personnel (hospital environment) who have direct contact with the patient, as well as being of interest to public health specialists. Financial issues related to the occurrence of the above-mentioned micro-organism are being increasingly raised. Due to the scale of the phenomenon, we are beginning to pay attention to the significant system costs caused by the diagnosis and treatment of CDI infection and its complications. Studies indicate that the nosocomial infection of *C. difficile* complicates hospitalisation, by increasing the cost by more than half and extending patient’s stay by an average of 3.6 days. Material and methods: The aim of this study was to attempt to calculate the estimated costs associated with the prolonged hospitalisation of patients with nosocomial CDI infection, using the example of a hospital in Lodz. A total of 53 completed hospitalisations of patients treated in the period of January–August 2018 were analysed, during which hospital *Clostridioides difficile* infection was identified. For the purposes of this study, statistical data collected in the hospital’s IT system were also analysed, covering 44,868 hospitalisations in the Jan–Aug 2018 period, during which no hospital infection occurred. They was a control group, in which the analysed cases were compared. The obtained data in the study determined how long each patient with *Clostridioides difficile* infection stayed in the hospital (from the moment infection was diagnosed until the day of hospital discharge), and which diagnosis related groups (DRG) (according to National Health Fund guidelines) were assigned. The average length of patient stay without infection within a given DRG group in each hospital ward was also determined. The collected materials became the initial point for the final analysis of hospital costs and the length of hospital stay caused by *Clostridioides difficile* infection. Results: *Clostridioides difficile* infection extended the hospital stay by an average of almost 12 days. The average cost of prolonged hospitalisation due to CDI infection (according to the average cost per person-day) was about PLN 7148 (1664 EUR), which gave a total value of about PLN 378,860.6 (88,240.5 EUR) in the examined period. At the same time, the average expenditure from the National Health Fund for hospitalisation due to CDI infections increased by about PLN 6627 (1542.8 EUR), which in the analysed period translated into over PLN 351,232.0 (81,505.5 EUR) (according to settlements with the National Health Fund). The outcome indicates that there is a clear relation between CDI and the anticipated length of hospitalisation of patients without an infection.

## 1. Introduction

An asymptomatic presence of *Clostridioides difficile* (CDI) in the gastrointestinal tract has been established in almost 3% of healthy adults and up to 80% of healthy newborns [1]. The carrying of this micro-organism increases by 16–35% in people treated in hospitals, and increases in proportion to the length of the stay in the hospital and during antibiotic treatment [2].

The frequency of symptomatic *Clostridioides difficile* infections (estimated to be an average approx. 30%) increases with age. It mainly results from the presence of many risk factors in elderly patients, which leads to the perturbation of immunological balance and facilitates the development of infection [3]. The risk of complications and death also increases with age. It is estimated that 90% of deaths caused by *Clostridioides difficile* infection and its consequences occur in patients older than 65 years, and the known demographic trends and ageing population will not improve this situation.

*Clostridioides difficile*, as the main cause of infectious diarrhoea in hospitalised patients is a significant challenge to medical personnel (hospital environment) in direct contact with patients, and is the subject of interest for public health professionals. Knowledge of the frequency of infections, risk factors and methods of preventing infections is the initial point and necessary element for establishing strategies and standards for the management of patients suspected of a *Clostridioides difficile* infection. The ability to collect and use statistical/epidemiological data is the basis for correct management of the risk of hospital infections, as well as their associated costs. The economical aspect related to the presence of the aforementioned micro-organism is also starting to be mentioned more frequently. Due to the scale of the phenomenon, we are starting to notice significant systemic costs caused by the diagnostics and treatment of CDI infections and their complications. Studies indicate that the nosocomial infection of *C. difficile* complicates the hospitalisation, by increasing the cost by more than half and extending a patient’s stay by an average of 3.6 days.

Most healthcare systems worldwide cover all of the costs related to the treatment of patients admitted to healthcare facilities for *Clostridioides difficile* infections. However, not all of them compensate for the expense resulting from the complications of the hospitalisations themselves. 

In Poland, studies of the actual costs of hospital infection are practically non-existent. Hospitals financed with public funds (with contracts with the National Health fund, the payer) do not conduct such analyses because they are not required to. The hospital IT systems available on the market are not oriented to the collection of the information required for such calculations. This creates a problem in obtaining not only reliable and credible data on the subject, but any data on the subject. 

The goal of this study was to calculate the estimated costs related to the extended hospitalisation of patients with a nosocomial CDI infection.

## 2. Materials and Methods

The collected materials were obtained from one of the hospitals in the Lodz province. In total, 53 finished patient hospitalisations in the period of January–August 2018, during which a *Clostridioides difficile* hospital infection was identified were subjected to analysis. Twelve hospital wards were included in the study. Over the analysed period, a total of 438 cases of hospital infections occurred. All the identified cases were primary hospital infections, related to a healthcare facility. All infections were reported by the medical personnel to the hospital infections team operating in the facility, which appropriately verified them. The specimen used for tests was a liquid or unformed stool sample, collected from a patient with suspicion of CDI (new or unexplained cases of diarrhoea). Infection was confirmed by a test detecting *C. difficile* toxins, as a part of a multi-stage algorithm (Dehydrogenase GDH+toxins) [4]. 

Additionally, the person-day valuation at the wards where a *C. difficile* infection was identified was established (according to the accounting department’s calculations). It included, among others, the cost of stay, used medications and dressings, single-use medical equipment, performed laboratory and diagnostic tests, procedures and remuneration of medical personnel. As it was not possible to assign actual cost of stay to an individual patient (no evidence and cost account on the patient’s record), the presented person-day data were averaged.

For comparison purposes, an estimate was made of the hospital’s revenue related to the treatment of patients in whom a *C. difficile* infection occurred, and revenue from infection-free hospitalisations from a control group obtained from the National Health Fund (the public payer who covers the costs of hospital treatment). The healthcare system in Poland requires the entity, which provides medical services, to assign the patient to one of the diagnosis related groups (DRG), in order to finance the patient’s treatment. Assigning each patient to a specific DRG group allowed the segregation of analysed hospitalisations for their similarity. The DRG group is established based on specific diagnoses and procedures, which were performed during patient’s stay. Many of DRG settlement groups also take into account the criteria of a patient’s age and sex. The same method of treatment for the same illness will always be settled with the same group. This significantly facilitates the comparison of hospitalisations not only concerning the medical procedure, but also the generated costs. It also allows searching for non-standard stays, for example, differing significantly in the length of stay.

All the aforementioned statistical and settlement data are recorded in the IT system of every hospital financed by public funds, and are transmitted in an ongoing manner to the payer (NFZ). The costs borne by the hospital are then reimbursed based on the transmitted reports. 

It should be noted that NFZ finances the costs of CDI treatment only when the aforementioned is the basis for the patient’s hospitalisation. During stays where CDI is not the primary diagnosis, and it occurred as a complication during another specialised treatment, NFZ does not cover the costs of additional treatment of such an infection. Each extended stay is thus another financial burden for the healthcare entity. Such cases are discussed in this study (the infection occurred during hospitalisation, which was not of itself due to the infection).

The analysis of collected data enabled us to establish how long each *Clostridioides difficile* patient has stayed in the hospital; indicates the time from the moment that the infection was diagnosed until the day of hospital discharge; and to which DRG group he/she was assigned. The average length of stay of a patient without an infection within the given DRG group at each hospital ward was also established. Subtracting the length of stay of patients with an infection by an average length of stay of patients without an infection (within the same settlement group) enabled the estimation of the length of extended hospital stay of the patients from the first group. Additionally, the length of the extended stay was combined with the average cost of person-day, obtained from the accounting department. The aforementioned person-day cost for each ward was calculated, taking into account all direct and indirect costs related to the provision of medical services, divided by the number of person-days in the period in question (in this case 8 months). Direct costs included used medication, materials, single-use equipment, performed tests and the medical personnel employment costs. Indirect costs, among others, included the costs of power, water and sewage, gas, washing, transport and board. It should be noted that without any knowledge about the settlement groups and the manner of financing by the public payer (NFZ), we would not be able to establish an average duration of hospital stay within a specific DRG group. Therefore, calculating the extended stay would have a very high risk of error, or would even be impossible. It is important to note that a lengthy hospitalisation at a given ward would not have to be related to a lengthy stay, but could result from the standard method of treatment, e.g., in the case of large, complex procedures in which the treatment duration will be longer than the planned diagnostic stay.

The collected materials became the initial point for the final analysis of hospital costs and the length of hospital stay caused by *Clostridioides difficile* infection. They enabled the estimation of the burdens on the healthcare system related to CDI treatment, using the example of the examined facility.

All collected data were subjected to statistical analysis with the use of the Excel spreadsheet from Microsoft Office suite. 

## 3. Results

**Figure 1:**
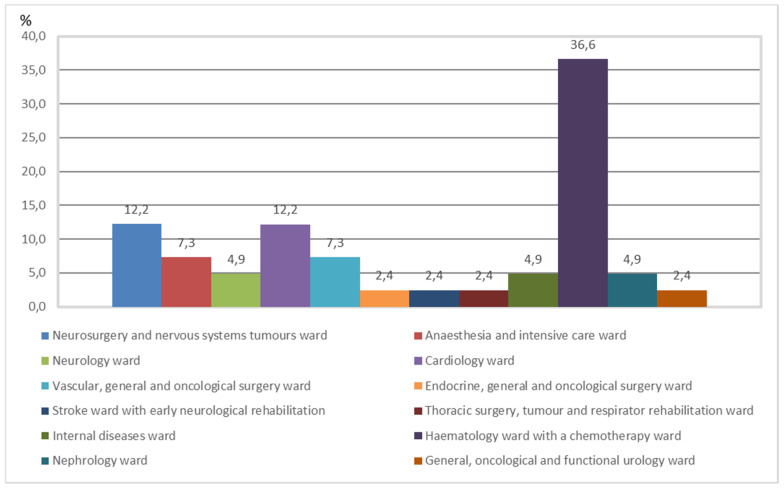
Occurrence of CDI infections in individual hospital wards (%).

The conducted analysis identified 53 hospitalisations complicated by a *Clostridioides difficile* infection, which amounted to 11.88%. The occurrence of CDI infections in individual hospital wards is presented in Figure 1.

For each of the 53 analysed CDI infection cases, the date of admission, the date on which the infection was diagnosed and the date of discharge and the DRG group were established. Based on this data, the average duration from the commencement of stay until the confirmation of CDI diagnosis was established—11.56 days (median—10 days, mode—1 day). The average duration of hospitalisation of patients with CDI from the moment of diagnosis until the treatment at the facility was finished (discharge) was also calculate—12.68 days (median—10 days, mode—4 days). 

Assigning each hospitalisation (both with infection and without infection, from the control group) to an appropriate DRG group enabled the estimation of the average time of treatment for patients with CDI and without an infection.

For each hospital ward based on the finished hospitalisations (without hospital infection, control group) an average stay duration and their median and modal value were established (Table 1). It can be easily seen that the specifics and character of the ward have an immense impact on the length of stay (type of ward frequently determines the average length of hospitalisation).

In Table 2, a statistical and settlement data fragment for all 44,868 hospitalisations under analysis finished in the period of I-VIII 2018r., to which a DRG settlement group was assigned and the value of financing acc. income from the National Health Fund. The collected data also enabled the establishment of a specific duration of stay, in days, for each hospitalisation from the control group (without hospital infections). On this basis, the average length of patient treatment was estimated, divided into diagnosis relate groups and average costs. 

Based on information obtained from the hospital’s financial department, each ward in which a CDI infection was identified in the given period was assigned an average person-day value, which amounted to (the national average in 2018, for comparison, amounted to 4585.03 PLN (the exchange rate for EUR 1 of 31.08.2018 amounted to PLN 4.2953)):

- General, oncological and functional urology ward—PLN 659.
- Internal diseases ward—PLN 668.
- Cardiology ward—PLN 836.
- Endocrine, general and oncological surgery ward—PLN 629.
- Vascular, general and oncological surgery ward—PLN 958.
- Neurology ward—PLN 683.
- Neurosurgery and nervous systems tumours ward—PLN 616.
- Thoracic surgery, tumour and respirator rehabilitation ward—PLN 926.
- Anaesthesia and intensive care ward—PLN 2245.
- Nephrology ward—PLN 660.
- Stroke ward with early neurological rehabilitation—PLN 639.
- Haematology ward with a chemotherapy ward—PLN 597.

The need to base the analysis on the averaged value of person-day specified above results from the fact that in the examined hospital, actual costs (used materials, antibiotics, examinations etc.) were not assigned to a specific patient. Therefore, it was not possible to indicate unequivocally the expenses related to the treatment of CDI infection.

Based on all collected data and calculations, we managed to establish the average extended duration of stay related to a *Clostridioides difficile* infection and related costs. They were estimated with two methods: by using information on the valuation of hospitalisation, according to settlements with the NFZ; and based on actual costs of a person-day at each ward. The most important results of the study are presented in Table 3.

## 4. Discussion

The issue of hospital infections is a subject in which specialists from various academic fields (including clinicians, epidemiologists or microbiologists) have exhibited a keen interest. Due to the cost associated with infection, CDI are drawing the attention of hospital directors and financiers. This is particularly if the infection causes a series of long-lasting, expensive and difficult to treat consequences and complications. Unfortunately, the number of published articles concerning the cost of CDI treatment in Poland remains small. This is possibly caused by the fact that many patients do not have a patient assigned cost account. Most of the collected articles are obtained from foreign language journals [5,6].

A study conducted in the United States [7] indicates that the frequency of diseases related to CDI infection has increased by approximately three-fold over a 9-year period (from 31/100.000 to 84/100.000) [8]. It was also estimated that around 300,000 cases are diagnosed in the US each year. Subsequent studies indicate that *C. difficile* infections are in 18th place, among the main causes of mortality in the >65 years age group [9]. At the same time, the costs of treatment of a single patient with a CDI infection amount from USD 2000 to 72,000 [10,11,12,13,14]. Another available publication on the economics of CDI infections in the US has indicated that this infection increases hospitalisation-related costs by more than half, and lengthens a patient’s hospital stay by an average of 3.6 days. It was also calculated that the median total duration of hospital stay in patients with a complication of a *C. difficile* diarrhoea was 7 days longer than in patients without the diarrhoea. At the same time, annual expenditures related to diarrhoeas caused by an infection with this bacteria fluctuate around USD 1.1 billion [15]. Similar data are presented in the study by McGlone et al. [16], who, in their calculations in addition to expenditure directly related to the treatment, have also include the societal costs of the infection, e.g., losses related to the patient’s inability to work during the course of the disease. They indicated that an average patient’s treatment cost borne by the hospital amounted from USD 9179 to 11,456, for the payer financing the stay the amount fluctuated within the range from USD 8932 to 11,679, and the social costs amounted from USD 13,310 to 16,464. The model proposed by the authors has estimated that the annual economic burden of CDI in the US would amount to USD 496 million for the hospitals, USD 547 million for the payer and USD 796 million for society. All of the arguments presented above confirm unequivocally that *C. difficile* infections are indeed expensive both for the hospital, the payer and for the entire society [17].

The study by Gabriel and Beriot-Mathiot [18] has enabled the estimation of costs related to CDI infections and the time (duration) of the hospitalisation. The first estimation amounted to the following: USD 6774 to 10,212, in the case of a patient requiring hospital admission due to symptoms of an infection; USD 2992 to 29,000, in the case of the need to treat an infection acquired during the hospitalisation; and USD 2454 to 12,850, in the case when an unequivocal classification was not possible. The ranges of the latter amounted, respectively, to the following: 5–13, 2.7–21.3 and 2.8–17.9 days.

The problem of epidemics of infections caused by the discussed micro-organism does not occur only in the United States. Available (though scarce) publications also concern Europe [19,20], Asia, Australia and Central America [21,22]. Based on data published by the National Hygiene Department (NHD), the CDI incidence rate in Poland was 30.2/100000 (2018 year) [23]. In the years 2014–2018, the incidence rate of CDI in our country was constantly high: 16.7; 23.3; 30.4; and 30.2 in years 2014–2018, respectively [24]. According to data from European Clostridium Difficile Infection Surveillance (ECDIS) in Poland, the frequency of infections caused by this bacteria amounts to 76 cases per 10,000 hospital admissions, with the assumption that the average for hospitals in Western Europe amounts to 23 per 10,000 admissions [25,26]. Unfortunately, there are no studies concerning the cost of CDI infections. The most reliable and fact-based article on the cost of infections (without differentiating by the type of micro-organism) is the analysis conducted by a team of specialists from the Polish Association for Hospital Infections (38 entities have participated in the study). Based on the analysis, we know that the duration of hospitalisation of infected patients was in most cases longer than the average stay of the entire study population of patients. Patients with an infection were hospitalised for an average of 16.2 days, which compared to the average length of stay of the remaining patients amounting to 7 days meant an average extension of the hospitalisation of these patients by approximately 9 days. Similar outcomes were presented in an article published in 2016. This article indicated that there is a clear relationship between CDI and the anticipated length of hospitalisation of patients without an infection. The authors simultaneously state that CDI rates are easy to measure and report, and thus may provide an important marker for hospital efficiency and/or quality [27]. At the same time, annual costs of the treatment of all infections in three selected hospitals were calculated, amounting to PLN 1 185,824.0, PLN 593,220 and PLN 550,000. 

The amounts specified above seem enormous to the average citizen. However, taking into account the sometimes multi-million contracts of healthcare facilities, the specified values do not present a significant problem to every director/manager (thus the lack of interest in the subject noted in the study). It should be noted that summarising all the costs that are generated in Poland by healthcare facilities for the treatment of complications of infections and estimating the possible savings reaches unimaginable values, up to tens of millions of zlotys. Millions that could be used for the implementation of new, innovative techniques and treatment methods and increase the availability of services.

## 5. Conclusions

The problem of hospital infections, including *C. difficile*, is a subject that impacts not only the fields of procedures, standards and quality of provided services. This is a field in which an increasing frequency is associated not only with patient safety, but also with issues with an enormous impact on the area of the finances of healthcare entities. Extended hospitalisations, additional costs through providing non-standard, unanticipated treatment (including medication and diagnostic testing) and the risk of claims by the patients are the reasons for which hospitals more thoroughly and frequently look at losses caused by the treatment of infections. Restricted revenues in healthcare systems (strictly established limits in contracts with the payer, lack of additional financing of hospitalisations complicated by an infection) [17] with continuously increasing costs of operation require the managers of healthcare entities provide proper management of adverse events (including mainly hospital infections) and cash flows. 

Infections caused by *Clostridioides difficile* are becoming an increasingly serious problem for healthcare units because:

- A CDI significantly extends a hospital stay by almost 12 days.
- The average cost of extended hospitalisation due to a CDI infection (acc. to an average person-day cost) amounted to approx. PLN 7148 (1664 EUR), which amounted in total to approx. PLN 378,860.6 (88,240.5 EUR) over the examined period.
- Average NFZ expenditure for hospitalisations caused by CDI infections have increased by approx. PLN 6627 (1542.8 EUR), which in the examined period has resulted in a total amount exceeding PLN 351,232.0 (81,505.5 EUR) (acc. to the NFZ settlements).

## Figures and Tables

**Table 1:** Number and average time of hospitalisation (without identified infections) in hospital wards.

Ward	Number of Hospitalisations	Day Min.	Day Max.	Day Average	Day Median	Day Dominant
Endocrine, general and oncological surgery ward	1382	1	77	4.66	3	3
Thoracic surgery, tumour and respirator rehabilitation ward	1267	1	49	3.72	2	1
Vascular, general and oncological surgery ward	1396	1	100	4.69	3	2
Internal diseases ward	907	1	37	5.06	4	1
Haematology ward with a chemotherapy ward	5299	1	111	3.49	1	1
Anaesthesia and intensive care ward	199	1	130	18.06	12	1
Cardiology ward	996	1	47	4.77	4	3
Nephrology ward	441	1	77	7.22	6	3
Neurosurgery and nervous systems tumours ward	651	1	136	9.52	7	1
Neurology ward	1867	1	40	1.96	1	1
Stroke ward with early neurological rehabilitation	351	1	248	14.02	10	8
General, oncological and functional urology ward	1410	1	61	4.02	3	2

**Table 2:** Example/fragment list of hospitalisations, taking into account the duration of stay, DRG settlement group and its value (zlotys).

Number of Hospitalisation	The Duration of Stay	Value	DRG Group
1	1	0	HOSPITALISATION FOR OTHER REASONS
2	7	8220	L00 NEPHRECTOMY AND OTHER SIGNIFICANT OPEN KIDNEY SURGERY
3	3	1676	L72A PROCEDURES ON SCROTUM, TESTICLE, EPIDIDYMIS AND VAS DEFERENS > 17 YEARS OLD
4	3	1645.76	HOSPITALISATION IN CHEMOTHERAPY
5	72	70,199.49	T03 TREATMENT OF PELVIS AND THIGH IN MULTIPLE INJURIES WITH COMPLICATIONS
6	3	3948	K03 PROCEDURES ON THYROID AND PARATHYROID GLANDS
7	1	947.71	HOSPITALISATION IN CHEMOTHERAPY
8	3	3948	K03 PROCEDURES ON THYROID AND PARATHYROID GLANDS
9	3	3172	L26 MEDIUM ENDOSCOPIC SURGERY ON URINARY BLADDER
10	3	1676	F46 ABDOMINAL DISEASES
11	1	1427.8	Q66 VASCULAR DISEASES
12	6	15,582	M11 COMPLEX PROCEDURES ON UPPER REPRODUCTIVE TRACT WITHOUT COMPLICATIONS
13	3	3948	K03 PROCEDURES ON THYROID AND PARATHYROID GLANDS
14	1	1097	P12 OTHER GASTRO-INTESTINAL AND METABOLIC DISORDERS
15	1	541	HOSPITALISATION FOR OTHER REASONS
16	2	3533	G25E CHOLECYSTECTOMY > 65 YEARS OLD
17	2	7405.2	Q42G ENDOVASCULAR PROCEDURES-2. AND 3. GROUP
18	2	2914.52	HOSPITALISATION IN CHEMOTHERAPY
19	2	1741	L53 MEDIUM PROCEDURES ON URETHRA
20	8	6003	F32 MAJOR AND ENDOSCOPIC PROCEDURES ON THE LARGE INTESTINE
21	2	9180.6	Q44 ENDOVASCULAR PROCEDURES-4. GROUP
22	2	262.8	Q52 VASCULAR ACCESS IN RENAL REPLACEMENT THERAPY
23	7	2434	F07F OESOPHAGUS DISEASES < 66 YEARS OLD
24	4	2011.2	C13 MEDIUM PROCEDURES ON ORAL CAVITY, PHARYNX AND LARYNX < 18 YEARS OLD
25	4	6200.85	J02 COMPLEX PROCEDURES WITHIN THE BREAST
26	2	1575.88	ONCOLOGY HOSPITALISATION RELATED TO PROGRAMME PERFORMANCE
27	3	2011.2	C13 MEDIUM PROCEDURES ON ORAL CAVITY, PHARYNX AND LARYNX < 18 YEARS OLD
28	2	647	L64 MINOR PROCEDURES ON THE PENIS
29	4	16,233.6	H14 PRIMARY TOTAL HIP JOINT REPLACEMENT WITH BONE RECONSTRUCTION, HIP JOINT REPLACEMENT WITH THE USE OF METAPHYSEAL STEM, HIP JOINT CAPOPLASTY

**Table 3:** Assessment of the average extended duration of stay and costs related to a nosocomial *Clostridioides difficile* infection.

Parameter	Average	Dominant	Median	Min.	Max.
The average length of stay-the control group (without hospital infections)	12.15	29.81	13	1	44.43
The average length of stay-hospitalisation with infections	24.10	17	19	5	72
Extended stay (days)	11.95	11	8.6	−25.43	83.57
The average cost of extended hospitalisation (acc. to the NFZ) (PLN)	6627.03	1028.50	0.00	−25,371.90	146,301.16
The average cost of extended hospitalisation (acc. to person-day cost) (PLN)	7148.31	9196.00	6899.20	−57,090.35	51,479.12

## Data Availability

The data presented in this study are available on request from the corresponding author.
